# Critical librarianship in health sciences libraries: an introduction

**DOI:** 10.5195/jmla.2019.620

**Published:** 2019-04-01

**Authors:** Jill Barr-Walker, Claire Sharifi

**Affiliations:** ZSFG Library, University of California, San Francisco, San Francisco, CA, jillbarr@gmail.com; Gleeson Library, University of San Francisco, San Francisco, CA, cosharifi@usfca.edu

## Abstract

The Medical Library Association recently announced its commitment to diversity and inclusion. While this is a positive start, critical librarianship takes the crucial concepts of diversity and inclusion one step further by advocating for social justice action and the dismantling of oppressive institutional structures, including white supremacy, patriarchy, and capitalism. Critical librarianship takes many forms, but, at its root, is focused on interrogating and disrupting inequitable systems, including changing racist cataloging rules, creating student-driven information literacy instruction, supporting inclusive and ethical publishing models, and rejecting the notion of libraries as neutral spaces. This article presents examples of the application of critical practice in libraries as well as ideas for applying critical librarianship to the health sciences.

## INTRODUCTION

Critical librarianship (or critlib), rooted in critical theory, is about applying principles of social justice to our work in libraries. Critical librarianship, and critical theory in general, focuses on both critiquing and changing society as opposed to simply understanding or explaining it [1–3]. As Kenny Garcia demonstrated in his 2012 “Keeping up with...Critical Librarianship” article, critical librarianship challenges common perceptions and beliefs, everything from cataloging rules to information literacy instruction to archival materials [4].

This article builds on Garcia’s publication and provides a health sciences–focused update on critical librarianship. The literature surrounding critical librarianship addresses and problematizes the concept of libraries as neutral spaces, arguing that neutrality is both unachievable and harmful to oppressed groups. Without critically evaluating the work that librarians do and the services that libraries provide, libraries as institutions and librarians as professionals are supporting systems of oppression.

## WHY CRITICAL LIBRARIANSHIP NOW IN HEALTH SCIENCES LIBRARIES?

Although critical librarianship has been practiced in academic libraries and addressed in academic librarianship conferences and publications since at least 2005, health sciences librarianship has been slower to adopt this movement. In the past 100 #critlib Twitter chats, most of the topics were presented in an academic library context, with health sciences librarianship featured only once in a February 2017 conversation about “Business, professional, medical, health, & STEM education programs.” Public libraries, by comparison, were featured on three occasions [5].

Recent support for diversity and inclusion from the Medical Library Association (MLA)—including the creation of a Diversity and Inclusion Task Force, the election of MLA’s first Black president, and a host of themed events and talks at MLA ’18 [6–8]—indicate that health sciences librarians are ready to begin conversations about these issues. Recent hospital initiatives like federally mandated sexual orientation and gender identity data collection [9] and mandatory training around trauma-informed care and cultural humility [10] demonstrate an interest in these issues among the health care workforce. In order to keep up with the needs of our users, we must also learn about and engage in critical practice.

## HOW CAN HEALTH SCIENCES LIBRARIES APPLY CRITICAL LIBRARIANSHIP PRACTICES?

### Technical services and cataloging

Historically, cataloging, classification, and search engine algorithms have been presented as neutral, logical systems that do not reflect or reinforce societal biases. In reality, these systems at the foundation of information organization and retrieval do just that: reflect and reinforce a structure that privileges those who are male, cisgendered, heterosexual, white, and Christian [11]. Cataloging rules and subject headings can whitewash historical events, as in the indexing of material about the Japanese-American incarceration in concentration camps with subject headings like “Japanese Americans: Evacuation and relocation” [12]. Librarians who are engaged in critical practice have advocated for changes to classification schemes and controlled vocabularies as well as critical reflections on the subjective nature of cataloging and classification and a recognition that the language used in cataloging and classification is a reflection of broader social structures, including Christian hegemony, racism, and sexism [13].

Search algorithms reflect the biases, beliefs, and priorities of the companies that sell them and the programmers who write them; they are not just simple instructions [14, 15]. Search engines are entryways to information and can distort or make invisible entire populations. For example, Safiya Noble has written extensively on how Google’s racist and sexist algorithms retrieved dehumanizing, pornographic, racist results in response to the keyword search “Black girls” [15].

Health sciences librarians can encourage critical engagement with the catalog by teaching users not only to find items, but also facilitating activities like user tagging, which allows groups to identify and define their own populations. Health sciences librarians can also encourage patrons to examine the power structures at play when using corporate search engines, including Google and web-scale discovery tools.

### Library instruction

Several critical teaching methods have been used by library workers, including critical pedagogy, feminist pedagogy, and critical information literacy [16]. At their core, these methods prioritize learner engagement and personal agency and aim to create actors for social change rather than passive learners. Feminist pedagogy actively addresses patriarchal power structures by creating interactive, decentered classrooms [17] and critiquing traditional library assessment methods [18]. Additional scholarship in this area has included raising awareness of oppression and social issues via search examples and teaching exercises, and incorporating self-reflection into our teaching [16, 18].

As health sciences librarians, we can utilize critical methods in our teaching by rejecting the traditional lecture model and prioritizing activities and exercises that rely on learner input. Many of our users are health professionals or graduate students. We can take advantage of their expertise and clinical knowledge and work together to create class goals, learning outcomes, and search examples based on their existing knowledge and self-identified needs. Consciousness-raising through the use of social justice–related examples during instruction sessions is an easy way for health sciences librarians to employ critical pedagogy. Using search terms around coercive contraception or sexism in residency programs to demonstrate database search features can raise awareness about these issues and help create an inclusive classroom space. Because many health sciences library workers do not teach in traditional classrooms and instead lead in-service trainings, orientations, or meetings, we can be creative in applying these techniques by considering each interaction as a teaching moment.

### Reference and outreach

Critical reference is a framework that attempts to examine and reshape traditional library reference services from a simple question-and-answer interaction to a participatory experience that relies on users’ existing knowledge, interests, and needs [19, 20]. Critical reference practice can involve working with users to raise thoughtful questions about their research topics and reflect on the social impacts of those topics [21, 22], documenting the experiences of library workers of color at the reference desk [23], and using consciousness-raising to highlight problematic aspects of the search process, like sexist subject headings or the economics of academic research that lead to global inequities in database access.

Library workers apply a feminist ethic of care by interacting with our users as fellow humans: asking how users are feeling, acknowledging emotionally difficult research topics, creating spaces where users are comfortable interacting with library staff, and acknowledging the personal and affective nature of many reference interactions [21–23]. Like critical instruction, a key facet of critical reference is the empowerment of users. Similarly, critical methods of outreach move “beyond inclusion” to amplify the voices of marginalized groups and work with local communities to design library programming and services around their self-identified needs [24, 25].

During the 2016 MLA annual meeting, a panel was convened dedicated to advancing the conversation around the health information–seeking behaviors and needs of lesbian, gay, bisexual, trans, and queer (LGBTQ) patrons, with an aim to “advance the developing conversation on LGBTQ health sciences librarianship” and evaluate how health sciences librarianship is meeting those needs [26]. The panel highlighted the importance of providing culturally relevant service to LGBTQ patrons and recommended the creation of tool kits that contain concrete examples of how library workers can make spaces more accessible, including displays of visible signs of support and LGBTQ-specific resources like dedicated subject guides [26].

In health sciences libraries, we can also draw from the medical field to incorporate principles of patient engagement [27], shared decision-making [28], and narrative medicine in our provision of reference and outreach services [29]. These methods prioritize patient (or user) voices and require an understanding of the user’s needs in order to provide relevant information services. Taking time to ask our users about their interests in the information they seek can help turn impersonal, transactional interactions into “transformative” experiences that facilitate connections between personal experience and scholarly inquiry [21]. As with critical teaching methods, health sciences library workers can utilize this type of critical practice during each interaction with users.

### Scholarly communications

The promotion of social justice and application of critical librarianship principles to scholarly communications can take many forms. Academia, particularly at the higher ranks, is overwhelmingly (77%) white, with numbers of both faculty of color and women shrinking as rank increases [30]. Similarly, the publishing industry is overwhelmingly white and, at the executive leadership level, overwhelmingly male [30]. The same demographics are at play in librarianship. Together, the lack of diversity in these industries and environments limits the voices that are heard and the scholarship that is disseminated.

The provision of open access scholarly publishing platforms, such as library-managed institutional repositories, can provide a platform for authors who have traditionally been excluded from scholarly discourse communities. Scholarly communication librarians who perform outreach and education have used their platforms to educate students and patrons about inequities in scholarly publishing and the potential of open access publishing models to alleviate some of these inequities [30]. Educating the next generation of scholars about these issues, including information privilege or who has access to information, can “empower students to see themselves as agents of change, prompt discussion and reflection on how open access or closed scholarship impacts others, and pose questions to students about how they would like to share (or not share) their own work” [31].

Health sciences libraries, particularly smaller libraries or those in hospital settings, are uniquely situated to benefit from open access publishing models. However, gold open access, which is supported by author processing fees, is only an option for scholars who can afford to pay those fees. Library-based publishing, often through an institutional repository, has the potential to mitigate the harms associated with publishing in a capitalist economic system. Repositories allow providers in clinical settings without the large subscription budgets of academic medical centers to freely access publications and reports that have been uploaded.

Likewise, low-resource settings around the world gain increased access to scholarly research through repositories. Repositories have the potential to disrupt the for-profit, capitalist structures of traditional, subscription-based publishing. Furthermore, repositories that publish student and clinician work allow us to hear voices that are often excluded from scholarly conversations. Individuals with expert clinical knowledge, but for whom scholarly writing is an area of growth, will have greater opportunities to contribute to the body of knowledge in their disciplines when they can disseminate their work through a repository.

### Archives

Archives, like libraries, have historically consisted of materials focused on white history and, in particular, the historical acts and artifacts of wealthy, white, heterosexual men. In her 2018 article “Archiving While Black,” Ashley Farmer discusses both limitations in archival collections and frequent reactions of surprise and alarm that Black historians, scholars, and students receive when visiting archives [32]. Farmer recounts that she has more than once “looked up from [her] research to see paintings of white men famous for committing heinous acts against indigenous communities, or racist artifacts displayed proudly as if devoid of the context in which they were produced” [32].

Critical archival practices include ways to identify and dismantle white supremacy around description, appraisal, access or use, education, and professional life [33]. Other priorities involve including voices and stories of marginalized populations in archives, increasing accessibility of archival materials, deconstructing colonial archival terms (e.g., “collections” and “ownership”), rewriting offensive or racist description from the past, accessing digital cultural heritage materials according to cultural norms and instructions [34–36], and using a feminist ethic of radical empathy in archives or “a willingness to be affected, to be shaped by another’s experience, without blurring the lines between the self and the other” [37].

Medical history is full of acts that have oppressed and dehumanized vulnerable and marginalized groups, from forced medical experiments on Black people dating from the US slavery era to the twentieth century [38], to unethical contraceptive testing on Puerto Rican women in the 1950s [39], to forced sterilization of Native American women in the 1970s [40], to the treatment of gay HIV/AIDS patients in the 1980s [41]. In health sciences archives, we can acknowledge this legacy of racism and intersectional oppression in medicine, pay special attention to the materials of marginalized populations, and seek out those voices that are not always included in archival materials.

Health sciences librarians can also promote access to materials and curate displays that educate and engage our local communities around social justice issues. While providing access to archival materials is important, especially in hospital libraries that are not open to the public, we must also recognize the cultural sensitivities of some materials that demand limited access, for example, sexually explicit materials and materials that involve recorded cultural heritage of Indigenous groups [36, 42]. Partnering with relevant communities is not only essential to providing inclusive, conscious, and critical archival services, it is also an opportunity to garner community support and provide education about health sciences archives.

### Libraries and librarianship

It is important to recognize the structural racism built into our profession and our spaces. Just fifty years ago, libraries were segregated spaces. The myth of library neutrality that claims libraries and library workers are objective and that we should bring no biases or emotions to our work is challenged by critical librarianship, which promotes self-reflection among library workers, considers the effects of emotional labor [43], and advocates for the importance of offering equitable services to all users, which is in itself a rejection of the status quo. A critical evaluation of the field of librarianship includes examining the whiteness of existing diversity initiatives in librarianship [44], noting the dangers of considering our profession as a calling (or “vocational awe”) [45], and exploring feminist approaches to library leadership [46]. Such examinations attempt to disrupt the unjust status quo and the tenets on which librarianship is based.

MLA was founded in 1898 and officially integrated in 1939 after 10 years of deliberation [47]. MLA first publicly acknowledged the importance of “the elimination of racial prejudice” in health sciences librarianship in 1972, only 46 years ago [48]. Health sciences librarians must recognize that these are relatively new attitudes in our field; with 60% of health sciences librarians now over the age of 50, the majority of people in our profession were alive prior to MLA taking a public stand against racial prejudice, and one-quarter were born well before libraries were legally desegregated [49]. Acknowledging that health sciences libraries and library workers are not neutral is the first step in addressing broader issues in our organizations and profession. Health sciences librarians can critically evaluate our services and spaces and advocate for action to address inequities in our libraries, in our professional associations, and in the broader field.
